# Efficacy of acupuncture and moxibustion in adjuvant treatment of patients with novel coronavirus disease 2019 (COVID-19): A protocol for systematic review and meta analysis

**DOI:** 10.1097/MD.0000000000021039

**Published:** 2020-07-10

**Authors:** Qiongshuai Zhang, Xiaohong Xu, Shaoqian Sun, Fang Cao, Jiannan Li, Xun Qi, Guangcheng Ji, Yufeng Wang, Bailin Song

**Affiliations:** a Department of Acupuncture and Tuina,Changchun University of Chinese Medicine, Changchun; b Graduate school, Changchun University of Chinese Medicine, Changchun; c Department of Rehabilitation, China-Japan Union Hospital of Jilin University, Changchun; d Department of Acupuncture, The First affiliated Hospital of Henan University of TCM, Zhengzhou; e Department of TCM, Changchun University of Chinese Medicine, Changchun; f Department of Rehabilitation, The Third Affiliated Hospital of Changchun University of Chinese Medicine, Changchun; g Department of Tuina, Traditional Chinese Medicine Hospital of Jilin Province, Changchun, China.

**Keywords:** meta-analysis, systematic review, acupuncture, moxibustion, COVID-19, protocol

## Abstract

**Background:**

Novel coronavirus has infected 4.33 million people in more than 200 countries in the current global outbreak of COVID-19. However, there is still no effective drug to treat the disease, and acupuncture and moxibustion is utilized as adjuvant therapy for the treatment of COVID-19 in China.

**Methods:**

Nine electronic databases: PubMed, EMBASE, Cochrane library, Web of Science (WOS), Google Scholar, China National Knowledge Infrastructure (CNKI), Chinese Biomedical Literature Database (CBM), Chinese Scientific and Journal Database (VIP), Wan Fang database (Wanfang) and 2 clinical trials register platforms: Chinese Clinical Trial Registry (ChiCTR), ClinicalTrials.gov (www.ClinicalTrials.gov/) will be searched for RCTs of A&M for COVID-19. The screening process will be developed by 2 independent reviewers, and meta-analysis will be performed with RevMan (V5.3.5) software.

**Results:**

The study results will be contributed to a scientific journal after peer-reviewed for publication.

**Conclusion:**

The study will provide up-to-date evidence of the effectiveness and safety of A&M for patients with COVID-19.

**PROSPERO registration number:**

CRD42020185776

## 1 Introduction

Since December 2019, there existed a global outbreak of novel coronavirus disease 2019 (COVID-19) infected by novel coronavirus (SARS-CoV-2) which was named by the International Committee on Taxonomy of Viruses (ICTV) and other virologists.^[1]^ Extensive infectivity and strong pathogenicity of which has posed a great threat to public health,^[2–5]^ thus WHO put out a statement of a global public health emergency of COVID-19 On January 30, 2020. Epidemiological investigations have shown that main symptoms of COVID-19 are fever, dry cough, and fatigue, with a small number of patients suffering from nasal congestion, runny nose, sore throat, myalgia, and diarrhea.^[6–12]^ Most of the severe cases may rapidly develop into acute respiratory distress syndrome (ARDS), septic shock, refractory metabolic acidosis, multiple organ dysfunction syndrome (MODS), and hypoxemia within a week of onset.^[13]^ Globally, as of May 12, there were 4,098,018 confirmed cases, of which 283,271 deaths, reported to WHO.^[14]^ So far there is still no specific drug for COVID-19,^[15–17]^ due to the epidemic first broke out in China, acupuncture and moxibustion, which are traditional Chinese external treatment, have been used as adjuvant treatment for it in China. However, there has been no systematic review on the safety and efficacy of A&M in the treatment of COVID-19. Therefore, this study conducted a systematic review and meta-analysis on it by collecting literature, so as to provide evidence-based medical support for A&M treatment of COVID-19.

## 2 Methods and analysis

We will perform the systematic review according to preferred reporting items for systematic review and meta-analysis protocols (PRISMA-P) 2015 statement.^[18]^

### 2.1 Study inclusion criteria

We will include randomized controlled trials (RCTs) with A&M for COVID-19.

#### 2.1.1 Participants

Patients who were diagnosed with COVID-19, without limitation of age, gender, or racial.

#### 2.1.2 Interventions

The treatment schedule of the experimental group included acupuncture alone or moxibustion alone or both of the two treatments, and there is no restriction on the types or dosages of A&M.

#### 2.1.3 Comparisons

The control group will receive any kind of treatment without A&M (Western medicine, placebo or regular treatment).

#### 2.1.4 Outcomes

Outcome indicators include effectiveness indicators and safety indicators. Effectiveness indicators include primary outcome indicators and secondary outcome indicators. The main outcome indicator was the total clinical response rate, and the diagnostic criteria included Cured: the disappearance of the main symptoms, the normal body temperature, the disappearance of lung rales, the re-examination of chest X-ray showed the absorption of lung lesions, and the return of white blood cell count to normal; Effective: the main symptoms were relieved, lung rhombus was improved, chest X-ray showed that the lung lesions were not completely absorbed, and the white blood cell count was improved; Invalid: no improvement or aggravation of symptoms, signs, chest X-ray examination and abnormal white blood cell count. Total clinical effective rate = (number of cured cases + number of effective cases)/total number of cases ×100%;

Secondary outcome measures: antipyretic time, cough duration, rhombus disappearance time, imaging transition time, serum C-reactive protein level (CRP) after treatment.

Safety was referred to the incidence of adverse events (bleeding, pain, hematoma, syncope, etc.).

### 2.2 Search methods

We will search 9 electronic databases of PubMed, EMBASE, Cochrane Library, Web of Science(WOS), China National Knowledge Infrastructure (CNKI), Chinese Biomedical Literature Database (CBM), Chinese Scientific and Journal Database (VIP) and Wan Fang database (Wanfang) to identify literature of RCTs of A&M for coronavirus disease 2019 (COVID-19).

Besides, we will also search Chinese Clinical Trial Registry (ChiCTR) and ClinicalTrials.gov (www.ClinicalTrials.gov/) for in-progress trials with unpublished data. Table 1 shows PubMed search strategy.

**Table 1:**
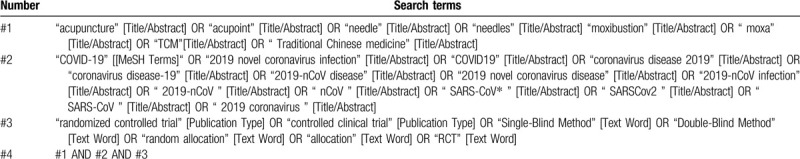
PubMed search strategy.

### 2.3 Data collection and management

#### 2.3.1 Selections of studies

**Figure 1:**
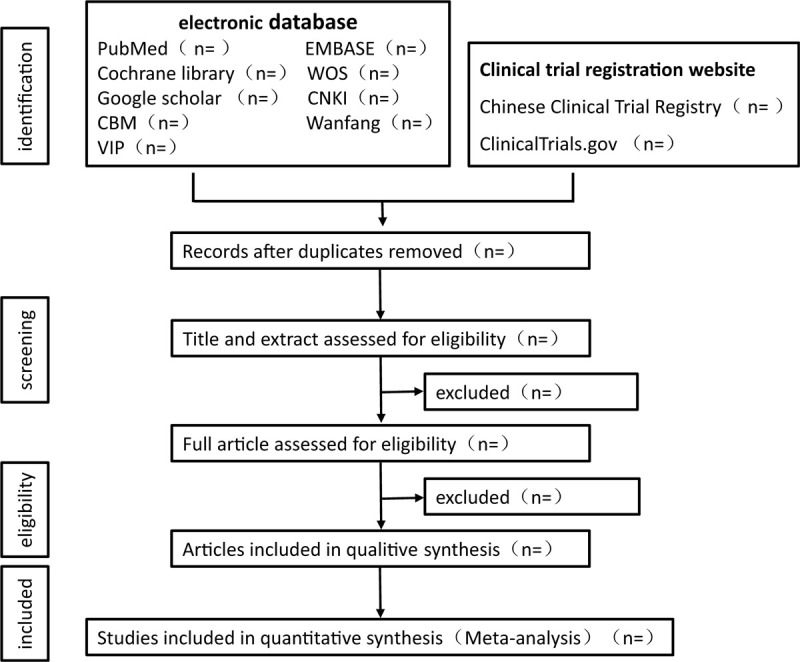
The screening process.

Two reviewers (JNL and XQ) will conduct studies selecting. First, they will eliminate duplicate articles with EndNote software (V. X9.0), they will screen literature with inclusion and exclusion criteria independently. Afterward, through reading titles and abstracts, literature that is obviously not applicable will be deleted. Finally, included articles will be chosen by screening the full articles. (Fig. 1 shows the screening process). All screening procedures will be undertaken by two researchers independently. Disagreements of decision making will be solved by referring to the third reviewer (BLS)

#### 2.3.2 Data extraction

Two reviewers (JNL and XQ) will select literature and extract data in accordance with the retrieval strategy. Title of the study, first author's name, publication year, journal; information of participants: gender, age, study design, sample size, diagnosis standard, intervention and outcome indicators will be extracted from the included studies with a standardized form for extracting data by the two reviewers independently from the included studies, they will also cross-check the results, disagreements will be solved by referring to a third reviewer (YFW).

### 2.4 Risk of bias assessment

Two independent reviewers (SQS and QSZ) will assess the risk of bias with Cochrane Risk of Bias Tool according to the Cochrane Handbook 5.1.0 for Systematic Reviews of Interventions. The 2 reviewers will assess 7 items, which consist of the risk of bias of sequence generation, allocation concealment, blinding of participants personnel and outcome assessment, incomplete outcome data, selective outcome reporting, and other bias. If there is disagreement during the assessing process, two reviewers will discuss or consult the third reviewer (XHX) for a decision. Three evaluation grades are low, unclear, and high risk of bias.

### 2.5 Measures of treatment effect

To assess A&M in the treatment of COVID-19. Pooled risk ratio (RR) with 95% credible intervals (CIs) will be used for investigating dichotomous variables. Standard mean differences (SMDs) with 95% CIs or weighted mean differences (WMDs) will be chosen for analyzing continuous variables.

### 2.6 Dealing with missing data

We will e-mail the corresponding author to obtain the necessary information, which is missing or insufficient. If failed, the analysis will be conducted based on the available studies, and we will review the potential impact of missing information.

### 2.7 Assessment of heterogeneity

I^2^ will be used for assessing statistical heterogeneity. It is acknowledged that I^2^ < 25% indicates negligible heterogeneity, 25% ≤ I^2^ < 50% indicates mild heterogeneity, 50% ≤ I^2^ < 75% moderate heterogeneity, and I^2^ ≥75% high heterogeneity.

### 2.8 Assessment of reporting bias

Over 10 studies included,^[19]^ we will take advantage of funnel plot to assess the reporting bias. Symmetrical funnel indicates no publishing bias, but if the funnel is not symmetrical, which indicates publishing bias exists. *P* value will be utilized, while less than 10 studies included.

### 2.9 Data syntheses

We will take advantage of RevMan software (version 5.3.5) for Statistical analyses performing. Only if there is no or mild significant heterogeneity (I^2^ < 50%; *P* > .1), we will apply the fixed-effect model, or the random-effects model will be selected.

### 2.10 Analysis of subgroups or subsets

If there exists potential heterogeneity, we will perform subgroup analysis based on methods of treatment, gender, age, or other items.

### 2.11 Sensitivity analysis

Robustness of the results will be assessed by sensitivity analysis performance which will focus on the processing method of missing data.

### 2.12 Grading the quality of evidence

Grading of recommendations assessment, development, and evaluation reliability study (GRADE) will be implemented to assess the quality of evidence. There are 4 levels of results: very low, low, moderate, and high.

### 2.13 Ethics and dissemination

Due to nothing of the information will be obtained from an individual participant, the systematic review does not need ethical approval.

## 3 Discussion

To our knowledge, there has not been a systematic review nor meta-analysis about A&M for COVID-19. A&M are traditional Chinese external medical treatments, which have been used in China for thousands of years. In ancient China, A&M were widely used in epidemic prevention and control.^[20]^ Early days of the outbreak in China, the world federation of acupuncture and moxibustion societies (WFAS) issued Guidelines on A&M intervention in COVID-19,^[21]^ recommending the use of A&M as adjuvant therapy for COVID-19. A&M has contributed to the prevention and treatment of COVID-19 in China. At present, China has made initial achievements in epidemic prevention and control, but the situation in other countries has been still grim. Therefore, we will systematically review the efficacy of A&M in the adjuvant treatment of COVID-19 and provide new ideas for the global fight against the epidemic.

## Author contributions

QSZ and BLS designed this study. JNL, XQ, and GCJ made the search strategy. FC and YFW designed the figure of the screening process. QSZ, XHX, and SQS wrote the manuscript, BLS and YFW edited it. All authors approved the publication of the protocol.

**Conceptualization**: Qiongshuai Zhang, Bailin Song

**Data curation:** Guangcheng Ji, Xun Qi, and Jiannan Li

**Formal analysis:** Qiongshuai Zhang, Yufeng Wang, Fang Cao, and Bailin Song

**Funding acquisition:** Bailin Song

**Resources:** Qiongshuai Zhang

**Software:** XunQi, Jiannan Li

**Writing – original draft:** Qiongshuai Zhang, Xiaohong Xu, and Shaoqian Sun.

**Writing – review & editing:** Bailin Song, Yufeng Wang
